# A Histopathological Feature of *EGFR*-Mutated Lung Adenocarcinomas with Highly Malignant Potential – An Implication of Micropapillary Element -

**DOI:** 10.1371/journal.pone.0166795

**Published:** 2016-11-18

**Authors:** Mai Matsumura, Koji Okudela, Yoko Kojima, Shigeaki Umeda, Yoko Tateishi, Akimasa Sekine, Hiromasa Arai, Tetsukan Woo, Michihiko Tajiri, Kenichi Ohashi

**Affiliations:** 1 Department of Pathology, Yokohama City University, School of Medicine, Yokohama, Japan; 2 Division of Respiratory Medicine, Kanagawa Prefectural Cardiovascular and Respiratory Center Hospital, Yokohama, Japan; 3 Division of Surgery, Kanagawa Prefectural Cardiovascular and Respiratory Center Hospital, Yokohama, Japan; 4 Division of Surgery, Saiseikai Yokohamashi Nanbu Hospital, Yokohama, Japan; Catalan Institute of Oncology, SPAIN

## Abstract

The purpose of this study was to define histological features determining the malignant potential of *EGFR*-mutated lung adenocarcinoma (LADC). Surgically resected tumors (*EGFR*-mutated LADCs with (21) and without (79) lymph node metastasis and *EGFR* wild-type LADCs with (26) and without (108) lymph node metastasis) and biopsy samples from inoperably advanced tumors (*EGFR*-mutated LADCs (78) and *EGFR* wild-type LADCs (99)) were examined. In surgically resected tumors, the *EGFR*-mutated LADCs with lymph node metastasis had the micropapillary element in a significantly greater proportion than others (Mann-Whitney tests *P* ≤0.026). The proportion of micropapillary element was higher in the *EGFR*-mutated LADC at the advanced stage (stage II, III, or IV) than in the tumor at the early stage (stage I) (Mann-Whitney test, *P*<0.0001). In the biopsy samples from inoperably advanced LADCs (177), *EGFR*-mutated tumors also had micropapillary element at a higher frequency than *EGFR*-wild type tumors (53/78 (68%), versus 30/99 (30%), Pearson x2 test, *P*<0.0001). In stage I *EGFR*-mutated LADCs (84), the tumors with the micropapillary element (34) exhibited a significantly higher recurrence rate than tumors without micropapillary element (50) (5-year Recurrence-free survival 64.4% versus 93.3%, log-rank test *P* = 0.028). The micropapillary element may be an exclusive determinant of malignant potential in *EGFR*-mutated LADC. It is suggested that *EGFR*-mutated LADC may develop through a distinct histogenesis, in which the micropapillary element is important for promoting progression.

## Introduction

Lung cancer is the leading cause of cancer-related death in the developed world, and lung adenocarcinoma (LADC) is the most common histological type of the disease. Recent research in molecular oncology has revealed that oncogenic mutations are required to promote tumor expansion, namely driver mutations, in LADC. These driver oncogenes include the *EGFR*, *KRAS*, *ALK*, *RET*, and *ROS* genes, mutations of which are mutually exclusive, and are crucial determinants indicating a favorable response to different molecular targeting agents [1] [2] [3] [4] [5] [6].

*EGFR* is the most common driver oncogene in LADCs, and mutations in this gene are seen in 20 to 50% of LADCs in Asians and 5 to 10% LADCs in Westerners [7] [8] [9]. *EGFR*-mutated LADCs have several unique features. They predominantly occur in females and non-smokers, and most cases are of the lepidic element-predominant histological subtype [7] [10] [11] [12] [13]. The lepidic element is a low-grade malignancy and is associated with a favorable outcome [14] [15] [16]. On the other hand, *EGFR*-mutated LADCs also include highly malignant tumors that are inoperably advanced. It remains unclear whether resectable tumors progress to become inoperable tumors or whether inoperable tumors develop independently through an exclusive carcinogenetic pathway. This is an important matter to be solved for better understanding of pathologic basis of *EGFR*-mutated LADC.

This study examined surgically resected tumors and biopsy samples from inoperably advanced tumors, and also defined the histopathological features associated with malignant potential in *EGFR*-mutated LADCs.

## Materials and Methods

### Patients

Three hundred and thirty-six LADCs that had been surgically resected (clinicopathological characteristics are presented in Table 1) and 177 LADC biopsy samples from inoperably advanced tumors (Table 2) were examined. These tumors were resected or biopsied between January 1997 and December 2013. Informed consent for the use of these samples for research purposes was obtained in writing. The ethics committees of Kanagawa Prefectural Cardiovascular and Respiratory Center and Yokohama City University approved the research plan.

**Table 1:** Clinicopathological characteristics of surgically resected lung adenocarcinomas.

	*EGFR*		
	Mutation (n = 142)	Wild-type (n = 194)	*P*-value
**Age (y/o)**			0.001*
** Median**	70.5	67	
** Range**	38–86	36–84	
**Gender**			<0.0001*
** Male**	47	135	
** Female**	95	59	
**Smoking status**			<0.0001*
** Never smoked**	89	48	
** Smoker**	53	146	
**Tumor size (mm)**			0.628
** ≤30 mm**	100	134	
** >30 mm**	42	60	
**Stage**			0.003*
** I**	103	106	
** II**	9	32	
** III**	29	51	
** IV**	1	5	

*EGFR*, *EGFR* mutation; y/o, years old; n, number of cases; *P*-values were calculated using the Mann-Whitney test (Age) and the Fisher's exact test (other subjects); Asterisk(*), statistically significant

**Table 2:** Clinical characteristics of inoperable lung adenocarcinomas.

	*EGFR*		
	Mutation (n = 78)	Wild-type (n = 99)	*P*-value
**Age (y/o)**			0.003*
** Median**	66	71	
** Range**	37–86	32–87	
**Gender**			<0.0001*
** Male**	25	74	
** Female**	53	25	
**Smoking status**			0.0002*
** Never smoked**	32	18	
** Smoker**	19	46	
** Unknown**	27	35	

*EGFR*, *EGFR* mutation; y/o, years old; n, number of cases; *P*-values were calculated using the Mann-Whitney test (Age) and the Pearson x2 test (other subjects); Asterisk(*), statistically significant

### Histopathological examination

Hematoxylin and eosin-stained sections were subjected to histological examination.

### Mutational analysis of the *EGFR* gene

The *EGFR* mutations (in exons 18, 19, 20, and 21) in surgically resected tumors were analyzed using previously described methods [17] [18]. The Scorpion amplification refractory mutation system method was used to search for mutations in the biopsy samples [19] [20].

### Statistical analysis

Pearson’s x^2^ test or Fisher’s exact test were used in combination with the Mann-Whitney test to analyze categorical and continuous variables, respectively. Recurrence curves were plotted using the Kaplan-Meier method and the absolute risk of recurrence at five years was estimated. Differences in the recurrence-free survival (RFS) were analyzed using the log-rank test. The Fleiss kappa statistic was used to measure interobserver agreement [21]. *P*-values of <0.05 were considered to be significant. All analyses were performed using JMP 9.0.2 (SAS Institute, Cary, NC, USA), SPSS version 21 (SPSS, Chicago, IL, USA), or the statistical software R (R Development Core Team 2014).

## Results

### Histological element that associates with malignant potential in *EGFR*-mutated LADCs

**Fig 1:**
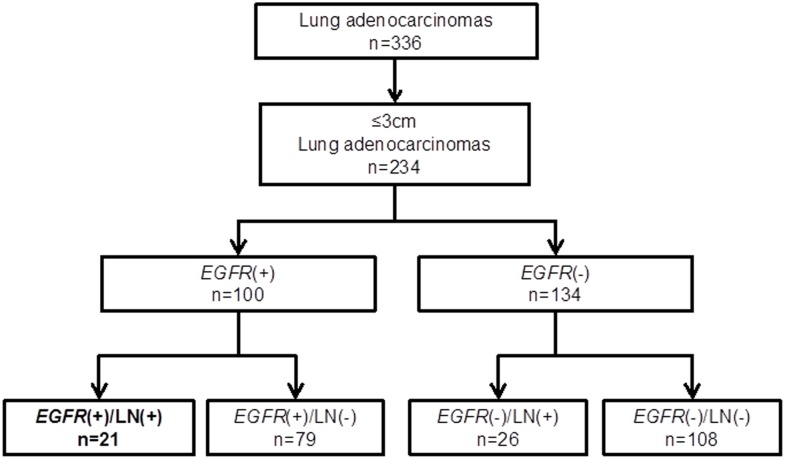
The flowchart used to assign the tumors to the four groups. n, number of tumors; *EGFR*, *EGFR* mutation; LN, lymph node metastasis; +, positive; -, negative.

**Fig 2:**
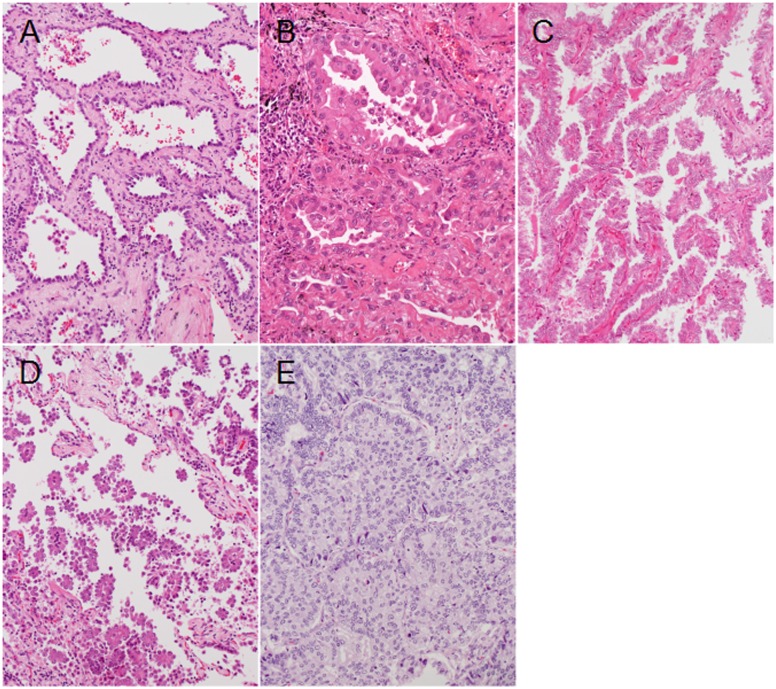
Representative appearances of the major histological subtypes of lung adenocarcinoma (hematoxylin and eosin stain, ×200). A, The lepidic subtype is characterized by the extension of neoplastic cells along the surface of the alveolar walls; B, The acinar subtype is characterized by tubular or glandular structures invading a fibrous stroma; C, The papillary subtype is characterized by the extension of neoplastic cells on the surfaces of fibrovascular cores; D, The micropapillary subtype is characterized by the formation of tufted papillary structures that lack a central fibrovascular core and float in the alveolar space; E, The solid subtype is characterized by the formation of solid nests consisting of neoplastic cells.

The study groups were assigned according to a flowchart described in figure 1 (Fig 1). Proportions of the histological elements (lepidic, acinar, papillary, micropapillary (mPAP), and solid elements) were described in 5% increments according to the World Health Organization (WHO) classification [22] [23]. The proportions in the *EGFR*-mutated LADCs with lymph node metastasis were compared with those in the other three groups. The proportion of mPAP element was consistently and significantly greater in *EGFR*-mutated LADCs with lymph node metastasis than in any of the other groups (Table 3). Differences in proportions of the other elements were not consistent in comparisons between *EGFR*-mutated LADCs with lymph node metastasis and the other groups (Table 3). Representative appearances of the elements are shown in figure 2 (Fig 2).

**Table 3:** Differences in the histological elements between the *EGFR*(+)/LN(+) group and the other groups.

	***EGFR*(+)/LN(+)**	***EGFR*(+)/LN(-)**	***P*-value**
**LEP**	30 (0–95)	70 (5–100)	0.0008*
**ACI**	30 (5–80)	10 (0–75)	0.023*
**PAP**	5 (0–60)	0 (0–80)	0.321
**mPAP**	5 (0–40)	0 (0–80)	0.025*
**SOL**	0 (0–70)	0 (0–30)	0.217
	***EGFR*(+)/LN(+)**	***EGFR*(-)/LN(+)**	***P*-value**
**LEP**	30 (0–95)	7.5 (0–80)	0.044*
**ACI**	30 (5–80)	32.5 (0–100)	0.554
**PAP**	5 (0–60)	0 (0–50)	0.009*
**mPAP**	5 (0–40)	0 (0–30)	0.026*
**SOL**	0 (0–70)	10 (0–100)	0.019*
	***EGFR*(+)/LN(+)**	***EGFR*(-)/LN(-)**	***P*-value**
**Lepidic**	30 (0–95)	80 (0–100)	0.013*
**ACI**	30 (5–80)	10 (0–100)	0.031*
**PAP**	5 (0–60)	0 (0–95)	<0.0001*
**mPAP**	5 (0–40)	0 (0–15)	<0.0001*
**SOL**	0 (0–70)	0 (0–95)	0.702

*P*-values were calculated using the Mann-Whitney test. Asterisk(*), statistically significant; *EGFR*, *EGFR* mutation; LN, lymph node metastasis; +, positive; -, negative; LEP; lepidic, ACI, acinar; PAP, papillary; mPAP, micropapillary; SOL, solid subtype

### The mPAP element and disease stage

**Fig 3:**
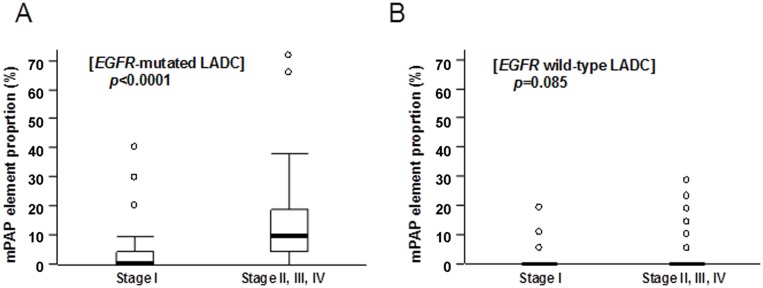
Proportions of the micropapillary (mPAP) element in different stages of surgically resected lung adenocarcinomas (LADCs). A, stage I *EGFR*-mutated LADCs (n = 103) versus (vs) stage II-IV *EGFR*-mutated LADCs (n = 39); B, stage I *EGFR* wild-type LADCs (n = 106) vs stage II-IV *EGFR* wild-type LADCs (n = 88); n, number of tumors examined mPAP element proportions are displayed as a box-and-whiskers plot with median (thick line), 25th to 75th percentile (box) and 10th to 90th percentile (whiskers) and outliers (circles). *P*-values were calculated using the Mann-Whitney test.

In *EGFR*-mutated LADCs, the proportion of mPAP element in the tumor at the advanced stage (stage II, III, or IV) was significantly higher than that in the tumor at the early stage (stage I) (Mann-Whitney test, *P*<0.0001; Fig 3A). In *EGFR* wild-type LADCs, the proportion of mPAP element showed no significant differences between the early stage tumors and the advanced stage tumors (Mann-Whitney test, *P* = 0.085; Fig 3B). These results suggested that the mPAP element may participate exclusively in the progression of *EGFR*-mutated LADC.

### The mPAP element in inoperably advanced LADCs

**Fig 4:**
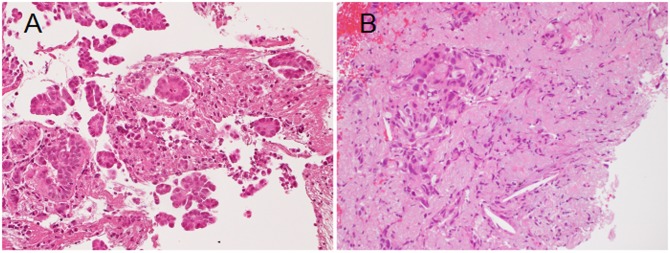
Representative histological appearances of the biopsy specimens (A, *EGFR*-mutated lung adenocarcinoma (LADC); B, *EGFR* wild-type LADC). The micropapillary element, which is composed of papillary structures lacking fibrovascular cores, floats in alveolar spaces (A, hematoxylin and eosin (HE) stain, ×200). The acinar element (and some crush artifacts) grows in collapse fibrosis (B, HE stain, ×200).

Biopsy samples from inoperably advanced LADCs were also examined. Representative histological appearances of the biopsy specimens are shown in figure 4 (Fig 4). The mPAP element was detected at a significantly higher frequency in *EGFR*-mutated LADCs than in the *EGFR* wild-type LADCs (53/78 (68%), versus (vs) 30/99 (30%), Pearson x2 test, *P*<0.0001). This result supports the idea that the mPAP element may participate exclusively in the progression of *EGFR*-mutated LADC.

### The mPAP element and postoperative recurrence

**Fig 5:**
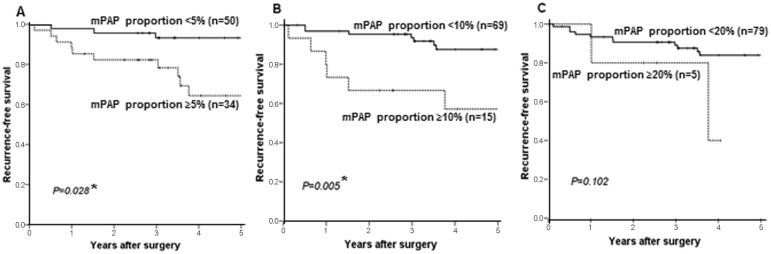
Kaplan-Meier recurrence-free survival curves of the association between the proportion of micropapillary (mPAP) element and disease recurrence in patients with stage I *EGFR*-mutated lung adenocarcinomas. A, tumors in which the mPAP element accounted for ≥5% of the tumor versus (vs) those in which the mPAP element accounted for <5% of the tumor (*P* = 0.028 in the Log-rank test); B, tumors in which the mPAP element accounted for ≥10% of the tumor vs those in which the mPAP element accounted for <10% of the tumor (*P* = 0.005 in the Log-rank test); C, tumors in which the mPAP element accounted for ≥20% of the tumor vs those in which the mPAP element accounted for <20% of the tumor (*P* = 0.102 in the Log-rank test); n, number of tumors examined; asterisk(*), statistically significant.

The association between the proportion of mPAP element and postoperative recurrence was analyzed in surgically resected stage I *EGFR*-mutated LADCs. The median follow-up period was 57 months (range: 1–159 months). Seventeen patients had recurrent disease and 15 patients died during follow-up. The recurrence-free survival (RFS) of *EGFR*-mutated LADCs that contained the mPAP element was worse than that of the *EGFR*-mutated LADCs that did not contain the mPAP element (Fig 5A). The difference was statistically significant when the mPAP element proportion cut-off value was set at 5% (5-year RFS 64.4% vs 93.3%, *P* = 0.028) or 10% (5-year RFS 57.1% vs 87.6%, *P* = 0.005) (Fig 5A and 5B), although no significant difference was found when the cut-off value was set at 20% (5-year RFS 40.0% vs 84.0%, *P* = 0.102) (Fig 5C). Number of tumors with mPAP element proportions of ≥20% may be too small for analysis. It was confirmed that the mPAP element could be a determinant of the malignant potential in *EGFR*-mutated LADCs.

### The potential prognostic impact of mPAP element for *EGFR*-mutated LADCs

**Fig 6:**
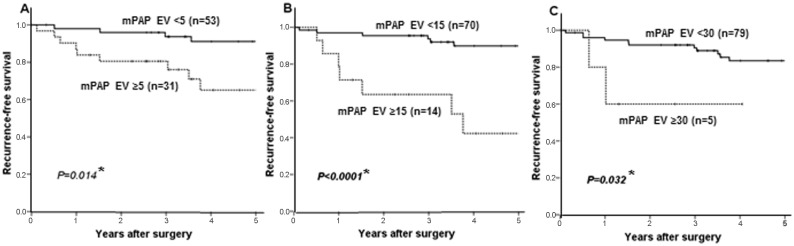
Kaplan-Meier recurrence-free survival curves of the association between the micropapillary (mPAP) estimated volume (EV) and disease recurrence in patients with stage I *EGFR*-mutated lung adenocarcinomas. A, tumors with mPAP EV of ≥5 versus (vs) those with mPAP EV of <5 (*P* = 0.014 in the Log-rank test); B, tumors with mPAP EV of ≥15 vs those with mPAP EV of <15 (*P*<0.0001 in the Log-rank test); C, tumors with mPAP EV of ≥30 vs those with mPAP EV of <30 (*P* = 0.032 in the Log-rank test); n, number of tumors examined; asterisk(*), statistically significant.

We additionally evaluated a prognostic impact of mPAP element for *EGFR*-mutated LADCs, as we considered an absolute volume of mPAP element may be more closely correlated with the malignant potential of the tumor than mPAP proportion. We defined the mPAP estimated volume (EV) as the percentage of the mPAP element multiplied by the square of the tumor’s largest radius [mPAP EV = (the tumor’s largest radius [mm])^2^ × (percentage of the mPAP element [%])/100]. The mPAP EV was found to be significantly correlated with RFS (Fig 6A, 6B and 6C). The lowest *p* -value (*P* <0.0001) was obtained when the mPAP EV cut-off value was set at 15 (5-year RFS 42.3% vs 89.9%; Fig 6B). Table 4 summarizes the univariate association between clinicopathologiacal factors and RFS. Lymphatic canal invasion (*P*<0.001), vascular invasion (*P* = 0.011) and mPAP EV (cut-off value: 15, *P*<0.001) were associated with worse RFS. Multivariate analysis revealed that the mPAP EV (*P* = 0.004) and lymphatic canal invasion (*P* = 0.009) were independent predictors of disease recurrence (Table 5). These results confirmed again that the mPAP element may be an important determinant of the malignant grade in *EGFR*-mutated LADCs. The mPAP EV also has a prognostic impact for predicting the postoperative recurrence of *EGFR*-mutated LADCs, which may be superior to the mPAP proportion (EV vs proportion, sensitivity 39% vs 33%; specificity 90% vs 86%; significance level <0.0001 vs 0.005, Figs 6B vs 5B). A Fleiss kappa statics from the mPAP EV (cut-off value: 15) judged by five pathologists supported good diagnostic concordance (Fleiss kappa value 0.689, *P* <0.001). The mPAP EV may be fit for clinical use.

**Table 4:** Clinicopathological characteristics and disease recurrence in patients with stage I *EGFR*-mutated lung adenocarcinomas (univariate analyses).

	n	%	5-year RFS (%)	*P*-value
**Sex**				0.683
** Male**	24	28.6	83.3	
** Female**	60	71.4	81.1	
**Age (y/o)**				0.656
** ≤65**	29	34.5	72.6	
** ≥66**	55	65.5	87.1	
**Smoking status**				0.55
** Never**	56	66.7	82.1	
** Former & current**	28	33.3	80.9	
**Surgical procedure**				0.176
** Lobectomy**	65	77.4	78	
** Segmentectomy**	7	8.3	85.7	
** Partial resection**	12	14.3	100	
**Tumor size (mm)**				0.152
** ≤30 mm**	61	72.6	84.7	
** >30 mm**	23	27.4	73.9	
**Stage**				0.098
** IA**	57	67.9	86.4	
** IB**	27	32.1	70.8	
**Adjuvant chemotherapy**				0.099
** No**	77	91.7	82.9	
** Yes**	7	8.3	71.4	
**Lymphatic canal invasion**				<0.001*
** Present**	4	4.8	75	
** Absent**	80	95	84.7	
**Vascular invasion**				0.011*
** Present**	19	22.6	59.6	
** Absent**	65	77.4	88.5	
**Pleural invasion**				0.252
** Present**	5	6	53.3	
** Absent**	79	94	83.9	
***EGFR* mutations**				0.611
** Major mutation (exon 19, 21)**	75	89.3	81.2	
** Minor mutation (exon 18, 20)**	9	10.7	87.5	
**mPAP estimated volume**				<0.001*
** <15**	70	83.3	89.9	
** ≥15**	14	16.7	42.3	

**Table 5:** Multivariate analysis performed using the Cox proportional hazards model.

	HR	95% CI	*P*-value
**mPAP estimated volume (cut-off: 15)**	6.274	1.78–22.17	0.004*
**Lymphatic canal invasion**	8.8	1.71–45.20	0.009*
**Vascular invasion**	0.949	0.238–3.78	0.940

HR, hazard ratio; CI, confidence interval; mPAP, micropapillary; Asterisk(*), statistically significant

### The mPAP element and types of *EGFR* mutations

No significant difference in types of *EGFR* mutations (major or minor mutations) between tumors with mPAP and those without mPAP was found (Table 6).

**Table 6:** Difference in types of *EGFR* mutations between tumors with mPAP and without mPAP element.

	tumors with mPAP element	tumors without mPAP element
**Major mutation (exon 19, 21)**	63 [52]	65 [24]
**Minor mutation (exon 18, 20)**	8 [1]	6 [1]

*EGFR*, *EGFR* mutation; mPAP, micropapillary;

The numbers of surgically resected tumors and [inoperably advanced tumors] are shown.

*P*-values were calculated using the Fisher's exact test.

*P*-values were 0.779 (surgically resected tumors) and 0.541 (inoperably advanced tumors).

## Discussion

**Fig 7:**
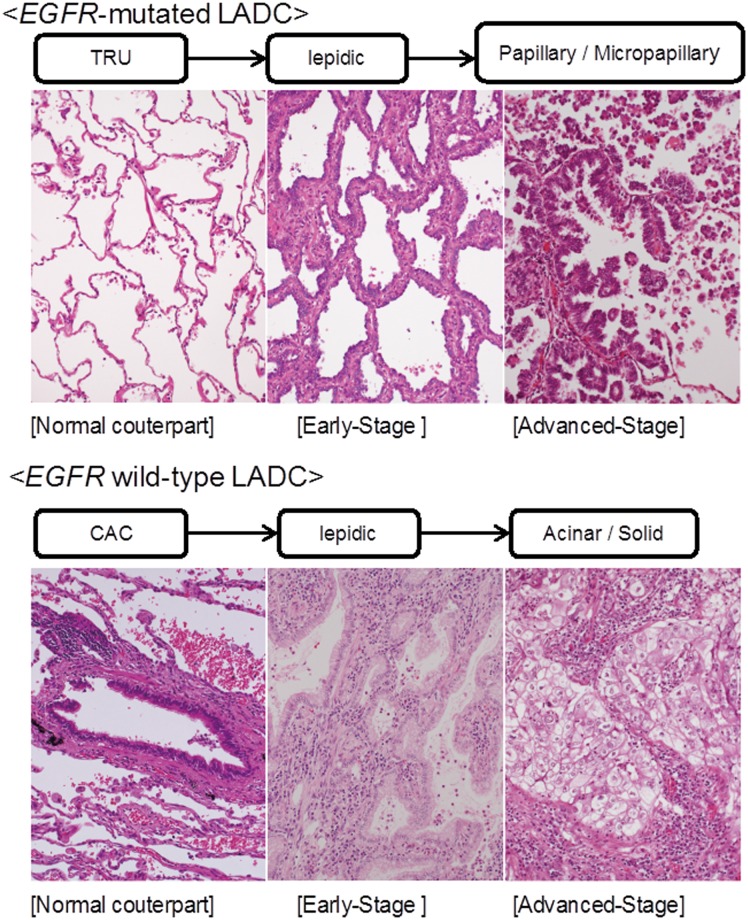
Hypothetical schema for histogenesis of the *EGFR*-mutated and the *EGFR* wild-type lung adenocarcinomas (LADCs). In early stages, *EGFR*-mutated LADC, which may develop from terminal respiratory units (TRU) [22], exhibits lepidic patterns consisting of neoplastic cells with hobnail or spheroid morphology. In advanced stages, they progress to form papillary and micropapillary patterns (upper panel). *EGFR* wild-type LADC, which may develop from the central airway compartment (CAC) [22], exhibits a lepidic pattern consisting of neoplastic cells with columnar morphology, and progresses to form acinar and solid patterns (lower panel). Magnification of all photographs is ×200.

The histopathological features of *EGFR*-mutated LADC have been extensively investigated [12] [13]. However, most studies examined only surgically resected tumors. The histological features of inoperably advanced *EGFR*-mutated LADC, which are really indicative for EGFR tyrosine kinase inhibitor (EGFR-TKI) treatment [22] [24], have not been defined. Thus, it is unclear whether resectable tumors progress to become inoperable tumors or whether inoperable tumors develop independently de novo. In this study, we examined both surgically resected tumors and biopsy samples from inoperable tumors and defined histological features determining the malignant potential of *EGFR*-mutated LADCs. The mPAP element preferentially arose in *EGFR*-mutated LADCs and was more common in advanced tumors. Previous studies have demonstrated that the mPAP element is associated with lymphatic canal involvement, leading to lymph node metastasis, which results in unfavorable LADC outcomes [25] [26] [27] [28]. Chao et al. recently reported that the mPAP element is associated with worse outcomes in patients with *EGFR*-mutated LADC, supporting our findings [29]. Taken together with these findings, *EGFR*-mutated LADC may develop through a unique carcinogenetic pathway in which the low-grade lepidic subtype progresses to the high-grade mPAP subtype (Schema shows the virtual carcinogenetic pathways of the *EGFR*-mutated and the *EGFR* wild-type LADCs; Fig 7).

On the other hand, it is noteworthy that the papillary element as well as mPAP element was also detected at a higher frequency in *EGFR*-mutated LADCs. This finding agrees with the notion that the papillary element may be a precursor for the mPAP element [30]. Papillary and mPAP are also occasionally found in *EGFR* wild-type LADCs, although these elements were rarely detected and their association with the malignancy grade was not statistically significant. Undefined mutations having potential biological activity equivalent to that of *EGFR* mutations (mutations of *EGFR* family members) may occur in *EGFR* wild-type LADCs with mPAP elements [31].

The present study also proposed that the mPAP EV may be a useful prognostic marker for predicting the recurrence of *EGFR*-mutated LADCs. Although patients with *EGFR*-mutated LADC generally exhibit favorable postoperative outcomes, a considerable proportion still dies of recurrent disease [12] [32]. Clinical trials of postoperative adjuvant EGFR-TKI therapy for patients with *EGFR*-mutated LADCs are currently in progress (WJOG6410L study, CTONG1104 study, ALCHEMIST study) [33] [34] [35]. The identification of tumors that are at high risk of recurrence and the adjuvant use of appropriate molecular targeting agents may be one way of improving postoperative survival. The mPAP EV parameter proposed here can be used to aid the identification of tumors that are at high risk of recurrence.

In summary, *EGFR*-mutated LADC may develop through a distinct carcinogenetic pathway, in which the mPAP element may play an important role in promoting progression. The mPAP element also has prognostic value. We hope that our efforts will increase current knowledge about the carcinogenesis of *EGFR*-mutated LADC and lead to improvements in the therapeutic strategies for such tumors.
